# Bio-inspired building blocks for all-organic metamaterials from visible to near-infrared

**DOI:** 10.1515/nanoph-2022-0690

**Published:** 2023-01-20

**Authors:** Samuel Thomas Holder, Carla Estévez-Varela, Isabel Pastoriza-Santos, Martin Lopez-Garcia, Ruth Oulton, Sara Núñez-Sánchez

**Affiliations:** Quantum Engineering Technology Labs, University of Bristol, Bristol, UK; CINBIO, Universidade de Vigo, 36310 Vigo, Spain; Natural and Artificial Photonic Structures and Devices Group, INL-International Iberian Nanotechnology Laboratory, Braga 4715-330, Portugal

**Keywords:** biomimetic, excitonic metamaterials, J-aggregates, organic metamaterials, photosynthesis, purple bacteria

## Abstract

Light-harvesting complexes in natural photosynthetic systems, such as those in purple bacteria, consist of photo-reactive chromophores embedded in densely packed “antenna” systems organized in well-defined nanostructures. In the case of purple bacteria, the chromophore antennas are composed of natural J-aggregates such as bacteriochlorophylls and carotenoids. Inspired by the molecular composition of such biological systems, we create a library of organic materials composed of densely packed J-aggregates in a polymeric matrix, in which the matrix mimics the optical role of a protein scaffold. This library of organic materials shows polaritonic properties which can be tuned from the visible to the infrared by choice of the model molecule. Inspired by the molecular architecture of the light-harvesting complexes of *Rhodospirillum molischianum* bacteria, we study the light–matter interactions of J-aggregate-based nanorings with similar dimensions to the analogous natural nanoscale architectures. Electromagnetic simulations show that these nanorings of J-aggregates can act as resonators, with subwavelength confinement of light while concentrating the electric field in specific regions. These results open the door to bio-inspired building blocks for metamaterials from visible to infrared in an all-organic platform, while offering a new perspective on light–matter interactions at the nanoscale in densely packed organic matter in biological organisms including photosynthetic organelles.

## 1 Introduction

Organic materials in nanophotonics have been used as organic semiconductors for applications such as organic light-emitting devices or solar cells [1]. Therefore, the design, development and materials processing have been focused on the optimisation of the absorption and emission spectral tuning of the material, and the transport and generation of charges within devices. Molecular J-aggregates have gained significant attention recently because they present an extremely narrowband response at the same time that they can be incorporated into optoelectronic devices such as photodiodes and detectors [2, 3]. This narrowband absorption can be tuned from visible to near-infrared by just selecting the right molecular species [4], opening the door for organic nanophotonics with an adaptable response in the visible or near infrared by just molecular species selection. But J-aggregates and their properties have not yet been explored as versatile building blocks for polymeric all-organic metamaterials.

Polymers have been introduced in metamaterials and nanophotonic structures in two implementations; as active materials which modify the response of an inorganic metamaterial [5, 6] and as active media whose properties are modified by an inorganic metamaterial [7]. In both cases, the polaritonic response is provided by the inorganic metamaterial while active properties are provided by the polymeric material. Recently, all-polymer metasurfaces have been developed including conducting polymers with switchable plasmonic resonances in the short/mid-wavelength infrared [8–10]. But, there are no reported examples of polymeric materials showing at the same time polaritonic properties which can be used to control light at the nanoscale by metamaterial structures while modifying the emission and absorption properties of their molecular constituents. In this work, we present an all-organic polaritonic library of polymer materials suitable as metamaterial building blocks with properties tunable from the visible to the infrared by selection of the molecular constituents. This will be achieved by taking inspiration from biological systems, building a family of water-based polymers doped by synthetic chromophore systems bio-inspired in photosynthetic antennas [11] and exploiting light–matter interactions at the nanoscale inspired by their arrangements at the nanoscale [12]. Furthermore, the intrinsic optical properties depending on the optical doping will open a new avenue for metamaterials design with supramolecular J-aggregates presented in a large variety of molecular families [4] (e.g. squarines [13], porphyrins, perylene bisimides [14], cyanines [15, 16], etc.) ranging from visible to near-infrared. Hence, this paper presents a bio-inspired polymeric platform based on J-aggregates which will break the glass ceiling of all-organic implementations in metamaterials in a sustainable, flexible, low cost, low weight and free of metals platform.

Natural biological systems have evolved complex structures at the molecular level which have been carefully optimised for specific functions. This complexity can be observed, for example, in membrane proteins where chemical selectivity is achieved through specific molecular key/site pairs [17], or in the arrangement of chromophore molecules in photosynthetic complexes (PCs) [18]. These PCs are composed of supramolecular chromophores contained in well-defined nanostructures where proteins play the role of responsive scaffolds [19–23]. These molecular arrangements have evolved to optimise light capture and exciton transport between chromophores, with proteins modulating energy pathways between them depending on sun irradiation conditions [24]. A significant amount of prior work has focussed on the investigation of PCs as absorbers and emitters by ultrafast spectroscopy and quantum modelling [12, 25]. However, these studies neglect the optical properties of the photosynthetic matter and hence, the potential photonic modes supported by the molecular nanostructures. In this work, inspired by the composition of the natural photosynthetic nanostructures of light-harvesting complexes (LHCs) of purple bacteria, we build up a library of bio-inspired organic matter which we use to model the light–matter interaction of photosynthetic nanostructures as all-organic building blocks for metamaterials.

LHCs of purple bacteria are composed of organic matter with a high concentration of dye molecules without presenting quenching (0.5–0.6 M) [11, 26]. They show annular structures composed of densely packed molecular aggregates of *π*-conjugated organic molecules with blue- and red-shifted bands (bacteriochlorophylls, phycocyanabilins, carotenoids, etc.) [26–29]. These supramolecular assemblies form a natural J- and H-aggregates with an exciton delocalised across monomers. Previous research on light-trapping and exciton transport strategies in natural systems typically analyses photosynthetic matter as an effective dielectric medium with a dispersive value of the refractive index. This definition of a macroscopic effective index of the membranes has furthermore been used in advanced models that describe proteins within photosynthetic complexes [20, 30]. While this may be an appropriate approximation for materials mainly composed of collagen or cellulose, photosynthetic LHCs nanostructures of purple bacteria are composed of densely packed H- and J-aggregates with strong absorptions and delocalised excitons which can promote significant modification of the local refractive index, with important consequences for how light interacts with these organic nanostructures [11, 26, 31, 32]. For example, our previous work demonstrates that thin films composed of densely packed J-aggregates within a polymer have strongly modified optical properties, achieving negative values of the real part of the electric permittivity and being able to support surface exciton polaritons (SEPs) [15, 33]. Here we demonstrate that these polaritonic properties are not unique to a single specific molecular compound. On the contrary, the polaritonic character can be achieved with a whole catalogue of molecules across the VIS and NIR spectral range, mirroring the broad range of chromophores appearing in photosynthetic organisms. These polaritonic properties arise from the presence of strong electric dipoles from delocalised Frenkel excitons in the constituent J-aggregates which are embedded within the polymer matrix. As LHCs can be composed by densely packed J-aggregates within protein scaffolds, we propose that the unusual optical properties required for SEPs may exist in the matter forming LHC nanostructures. Therefore exciton-polariton modes could play a role in the well-defined nanostructures of LHCs [34–36] of purple bacteria, with structural similarities between these natural nanoscale systems and nanoscale metamaterial building blocks [37, 38].

To investigate the physics of polaritons in LHCs of purple bacteria, we study the light–matter interactions of LHC architectures at the nanoscale through electromagnetic simulations, using as models the architecture of the LHC-2 of *Rhodospirillum molischianum* bacteria, together with the optical properties of a library of organic materials composed by artificial J-aggregates [11, 39, 40]. We would like to remark that natural light harvesting systems have a higher complexity than the model system proposed here. Natural light harvesting systems present combinations of several chromophores in one LHC where the delocalisation of the exciton can be dynamically controlled by the protein scaffold. Our model aims to show that in an instant of time, the exciton delocalisation over the chromophores forming the LHC, promotes the photon interaction with the LHC as a wave, confining the light at the nanoscale and enhancing light–matter interactions at wavelengths different than the intrinsic absorption of the chromophores. Thus, our simulations revealed that LHC-2 structures composed of densely packed J-aggregates acting as nano-resonators can confine light at the nanoscale at shorter wavelengths than the absorption of the bulk material. The optical response obtained depends strongly on the polarisation of the incident light, revealing that when the incident polarisation is contained in the photosynthetic membrane, the electric field is concentrated in the centre and edges of the nanorings, enhancing interaction with the reaction centre and promoting the coupling between closed LHCs. This offers a new perspective on the understanding of light–matter interactions in natural photosynthetic complexes of purple bacteria, demonstrating how supramolecular nanostructures of densely packed J-aggregates can shape and optimise the interaction of light and transport of energy within photosynthetic organelles. Understanding this mechanism could boost light-harvesting efficiency in metamaterial devices through a new family of molecular materials that mimic the carefully optimised molecular arrangement, concentration, and architectural design of natural photosynthetic systems, with J-aggregate and polymer materials forming the building blocks of such a platform.

## 2 Results

### 2.1 Bio-inspired polaritonic library

**Figure 1:**
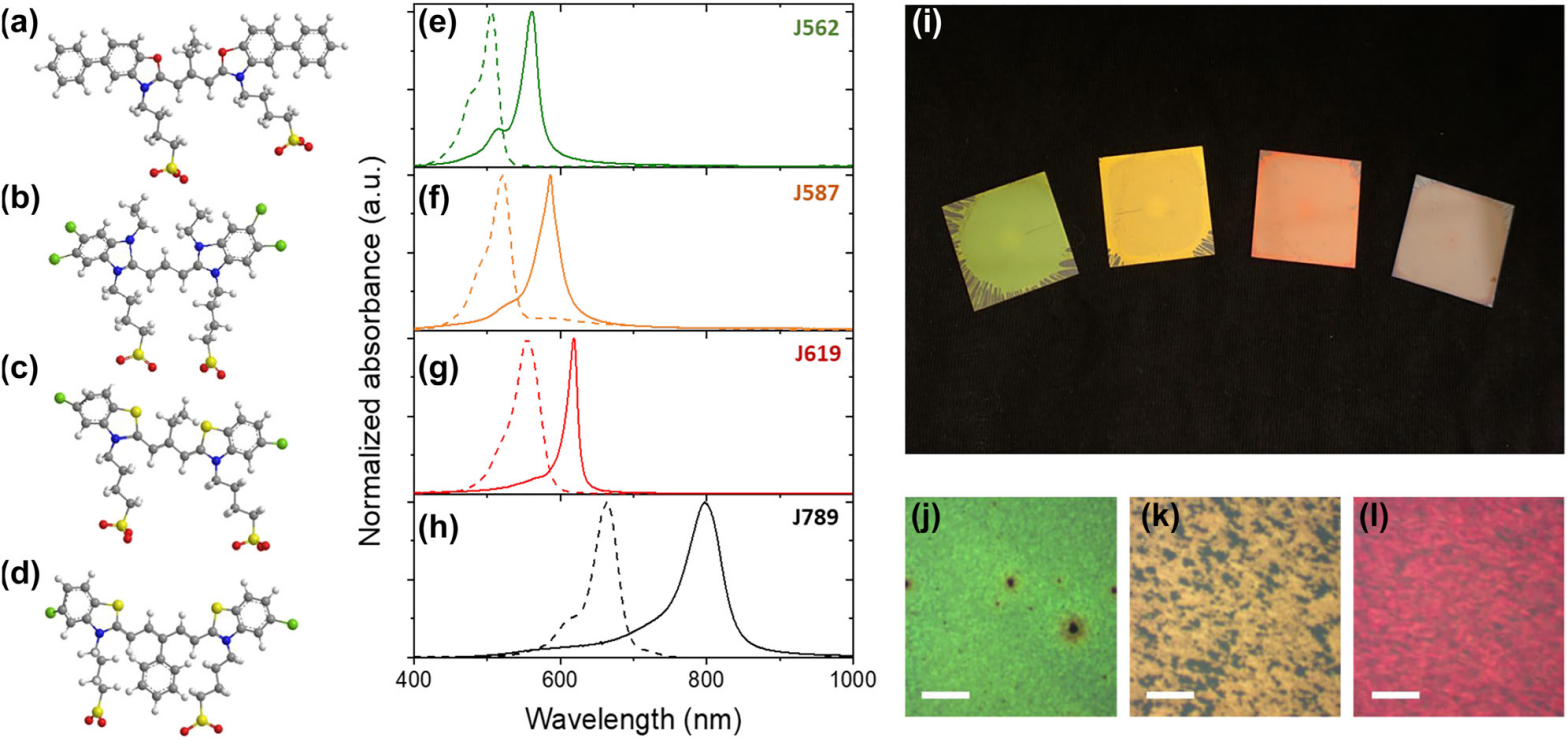
Bio-inspired polaritonic library. (a)–(d) Molecular structure of the monomers of the selected cyanine library for this work. They are named here according to the absorption peak of the J-aggregate conformation (chemical structures with higher resolution can be found in Figure S1). (e)–(h) Normalized absorbance of monomeric dye solutions in ethanol (doted lines, 100 μM) and J-aggregate:PVA mixtures (solid line) for the J-aggregates (e) J652, (f) J587, (g) J619 and (h) J789. (i) Picture of the J-aggregate:PVA films on top of black cardboard. From left to the right: J562, J587, J619 and J789 films. Optical microscope images of a 250 μm square area for dye (j) J562 (k) J587 and (l) J619 measured in reflectance microscopes under Köhler Illumination configuration. The scale bar is 50 μm.

To create a material consisting of both passive and active optical components, in analogy with proteins and chromophores in natural light-harvesting systems, we mixed J-aggregates with a water-based polymer. This physically separates the J-aggregates giving robustness to the final bulk photosynthetic-mimetic material. We controlled the conformal arrangement of the molecular aggregates to promote self-assembly into J-aggregates by increasing the dye concentration in final dye-polymer water solutions [4]. As a library of J-aggregates, we used commercial water-soluble closed-chain cyanine dyes with optical responses from the visible to the infrared. The structure of the monomers of the four J-aggregates selected for this work is shown in Figure 1(a)–(d): J562, J587, J619 and J798 (see methods for complete name). The Figure 1(a)–(d) shows the extinction of the monomer obtained from the dye solution in ethanol for the four cyanine dyes with peaks at 504 nm (J562), 519 nm (J587), 554 nm (J619) and 664 nm (J562). For a water-based polymer we selected poly (vinyl alcohol) (PVA) with a molecular weight of 85,000–124,000. The four cyanine water solutions (25 mM) were mixed with 6 wt% aqueous PVA solution (3:1 volume ratio) as previously described [15]. As shown in Figure 1(a)–(d), all four cyanine-PVA solutions show a narrow absorption peak red-shifted with respect to the peak of the monomeric dye that confirms the formation of J-aggregates. The J-aggregate:PVA films were prepared by spin-coating (10,000 rpm) of dye-PVA solutions on glass substrates obtaining a final mass ratio of J-aggregate to PVA in the bulk material of around 1:1.

**Figure 2:**
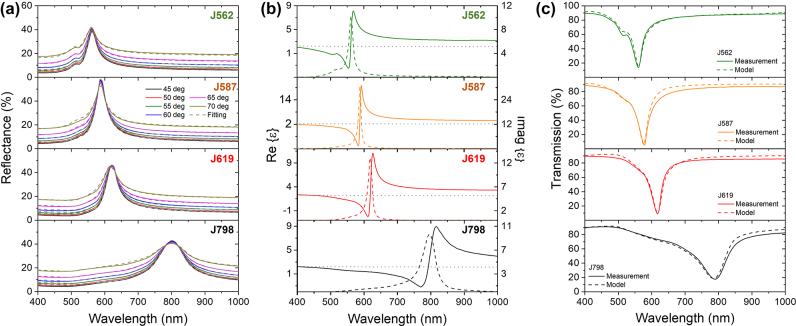
Optical response and optical properties of J-aggregate:PVA films. (a) Measured (solid lines) and fitted data (dashed lines) of un-polarised reflectance of J-aggregate:PVA films at an angle of incidence of 45, 50, 55, 60, 65 and 70°. (b) Real (solid line) and imaginary (dashed line) parts of the permittivity for the different J-aggregate:PVA films as indicated obtained by fitting the unpolarised reflectance data only. Real part of the permittivity of PVA from published data (grey dotted line) [42]. (c) Experimental and modelled transmission through the polymer films using the optical properties obtained from reflectance and the thickness from AFM. Panels from top to bottom: J562, J587, J619 and J789 films.

Figure 1(i) shows a picture of the four samples obtained. All the samples show a metallic lustre, each in a different spectral range, observed by the naked eye and through a microscope (Figure 1(j)–(l)). This high reflectance has a well-defined, vivid colour, because it is associated with a narrow region of negative real electric permittivity created by the intense absorption of the densely packed J-aggregates making up each film [15]. While metals generally have negative real electric permittivity in a broad spectral range, and therefore are reflective in a broad spectral range, these J-aggregate based materials exhibit this behaviour in a narrow spectral range, located at shorter wavelengths than the J-aggregate absorption, giving them a vividly coloured reflectance. Reflectance close to 60% is achieved for all four samples (see Figure 2(a)). Besides, the spectral response of the reflectance band is independent of the angle of incidence, demonstrating that it is inherent to the optical properties of the material and is not due to the creation of a Fabry–Perot cavity. The J-aggregate:PVA films were analysed by Atomic Force Microscopy (Supplementary Section 2). Table 1 shows the thickness and roughness of the different J-aggregate:PVA films. In general, the films are homogeneous on a 100 nm length scale. Regardless the dyes, the J-aggregate:PVA films present a roughness comparable to that of a metal film obtained by thermal evaporation (1–2 nm) [41] being the films with the largest roughness those obtained with J562 and probably due to randomly orientated bundles of J-aggregates at the film surface. For example, the J562 film with the largest roughness shows grain-like features (Figure S2(a)). Further work is required to investigate potential domain structure within J-aggrete:PVA materials. In this work we have assumed that the films are optically homogenous.

**Table 1:** Roughness (*σ*), thickness, polaritonic band, full half maximum width (FHMW) and minimum value of real part of permittivity (*ε*1(min)) of the J-aggregate:PVA films obtained with the different dyes.

Dye	σ (nm)	Thickness (nm)	Abs. peak (nm)	Polaritonic band^a^	FHMW (meV)^a^	*ε*1 (min)
J562	5.1 ± 0.3	31 ± 1.3	562	548–557 nm	55	−1.5
J587	3.2 ± 0.3	23 ± 0.8	587	552–587 nm	36	−8.3
J619	2.1 ± 0.1	38 ± 0.4	619	600–615 nm	49	−2.2
J798	2.0 ± 0.1	33 ± 0.7	798	763–775 nm	92	−1.2

^a^The polaritonic band is defined as the wavelength regions where the J-aggregate:PVA films are polaritonic (real part of permittivity <−1). Full half maximum width (FHMW) is the FHMW of the imaginary part of the permittivity.

**Figure 3:**
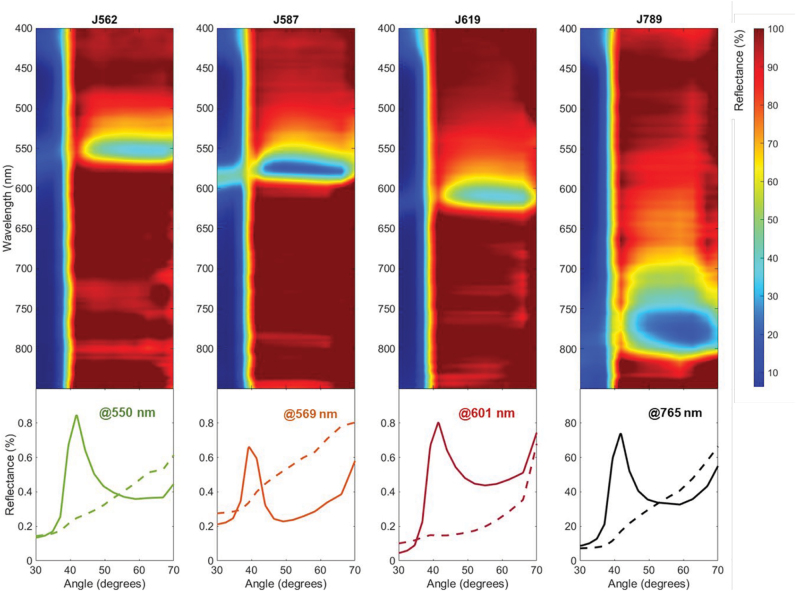
(The top panel) Measured p-polarised reflectance as a function of the wavelength and incident angle of J-aggregate:PVA films containing J562, J587, J619 and J798 J-aggregates, as indicated. (Bottom panel) Measured p-polarised and s-polarised reflectance as a function of the incident angle at the central wavelength of the polaritonic band for each film: 552 nm for J562, 569 nm for J587, 607 nm for J619 and 769 nm for J789, as indicated.

The optical properties of the films were estimated by fitting the unpolarised reflectance as a function of the incidence angle. The samples were modelled as a stack of two smooth layers: a thin J-aggregate:PVA film on top of a semi-infinite glass substrate. The thickness of the J-aggregate:PVA film was fixed to the measured value for each sample (Table 1). The resulting real and imaginary parts of the relative electric permittivity of each film are shown in Figure 2(b). To confirm the measured optical properties of each film, we measured the transmission spectrum through each sample and compared the experimental values with the modelled transmission spectrum (see Figure 3(c)). The good agreement between measured and modelled data demonstrates the accuracy of the obtained optical properties of each material. We would like to remark that the position of the minimum of the transmissions matches the absorption peaks of the J-aggregate:PVA mixtures (Figure 1(e)–(h)) confirming the presence of only J-aggregates in the spin-coated J-aggregate:PVA films. Each J-aggregate:PVA material achieves negative values of the real part of the relative electric permittivity. We have defined the polaritonic band of the material as the wavelength range where the real part of the permittivity is lower than −1 (see Table 1), where we expect the materials to support SEPs in the film-air interface. We would like to remark on the low values of real electric permittivity achieved, for example −8.3 in the case of J587 (see Table 1).

Based on the measured optical properties, each J-aggregate:PVA film is expected to support SEPs at the air-polymer interface within the polaritonic band. The top panels of Figure 3 show the p-polarised reflectance from 400 to 850 nm as a function of the angle for the four J-aggregate:PVA films measured under Kretschmann prism-coupling configuration by Fourier imaging spectroscopy (detailed description in S2). A dip in the p-polarised reflectance is observed for the four samples at larger angles than the critical angle for glass (41°). This dip in reflectance is observed in a narrow wavelength range corresponding to the polaritonic band specific for each constituent J-aggregate. The observed dips are broad in angle, suggesting that the SEPs supported by our J-aggregate:PVA films are affected by losses associated to the imaginary part of the permittivity. However, we would like to remark on the contrast between p- and s-polarisation measured for all samples, showing efficient mode coupling at central wavelengths of the polaritonic band (bottom panels of Figure 3).

In summary, all four J-aggregate:PVA materials show a wavelength-specific polaritonic response depending on the optical response of the constituent J-aggregate. Therefore, the negative permittivity region can be selected through the characteristics of the constituent J-aggregate, creating a library of plasmonic-like materials, with the potential to expand to a much broader range of molecules including cyanines and porphyrins. Table 1 shows the wavelength ranges where the J-aggregate:PVA materials exhibit real relative electric permittivity less than −1, where SEPs were excited. Table 1 also shows the full-width half maximum (FWHM) of the imaginary part of the permittivity and the minimum value reached. Note that broader optical resonances can yield negative permittivity if the dye in question reaches a sufficiently high density.

### 2.2 Building blocks inspired by purple bacteria light-harvesting complexes at the nanoscale

Considering that natural photosynthetic nanostructures of LHCs of purple bacteria contain densely packed J-aggregates, we hypothesise that these biological structures may also support SEPs, specifically local surface excitonic resonances (LSERs). These modes would be analogous to local surface plasmon resonances in plasmonic metamaterials, and we model the nanophotonic response of individual nanorings of densely packed dyes mimicking natural LHCs architectures as potential building blocks of an all-organic metamaterial platform, using the obtained optical properties of J-aggregate:PVA materials. Note that in comparison with natural LHCs, our model does not seek to exactly replicate effects such as optical losses, molecular disorder or coupling strength between the constituent monomers [43]. Moreover, molecular aggregates in natural light-harvesting complexes present defined molecular orientations which could lead to local refractive index anisotropies [26]. For simplicity, and following our observations of the materials developed in the experimental section of this work, we assume that the organic matter forming the nanoring shows isotropic optical properties. Instead, we are drawing an analogy between metamaterials and natural architectures of densely packed J-aggregates with important implications for the nanophotonics of the respective systems, including how light may be managed in natural photosynthetic supramolecular nanostructures through polaritonic modes mediating exciton transport or energy transfer [44, 45].

**Figure 4:**
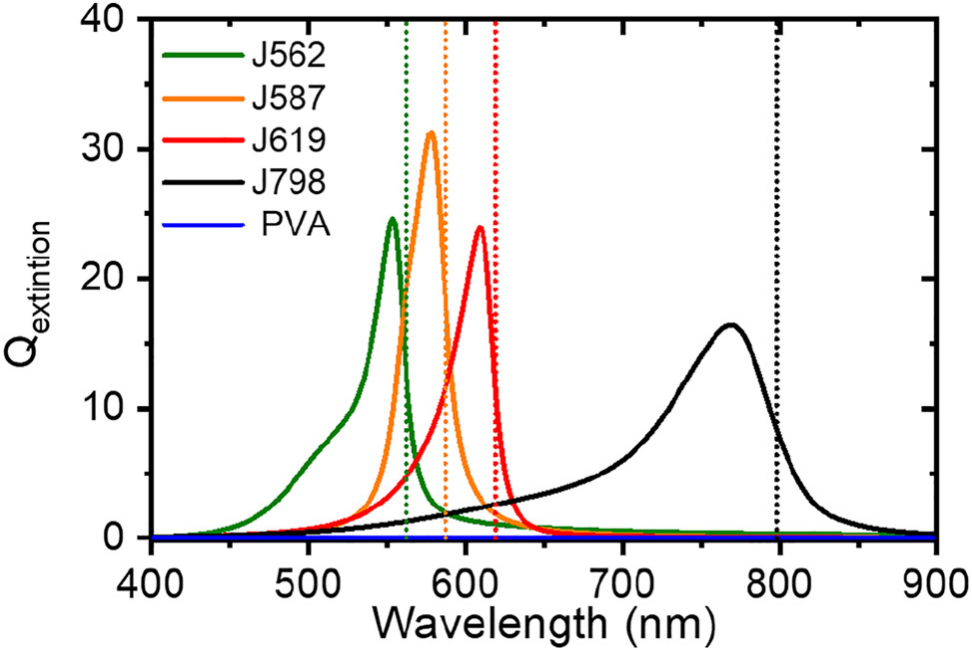
Normalised extinction efficiency cross section of simulated LH2 nanorings composed of only pure PVA (blue) and J-aggregate:PVA materials obtained via a combination of J562, J587, J619 or J798 with PVA. The coloured dotted line corresponds to the absorption peak of the bulk polaritonic materials (see Table 1).

There are a large variety of dye architectures described across the different photosynthetic systems in lamellar membranes, spherical vesicles or tubular structures [46]. But the most common feature of photosynthetic species of purple bacteria is a photosynthetic unit containing the reaction centre surrounded by peripheral dyes and proteins creating closed and open loop nanostructures. The size and dye distributions between species can change, but in the case of green and purple bacteria, LHCs show ring structures with a total external diameter varying between 5 nm [21] to more than 10 nm with some variations depending on the species [21, 29, 47, 48]. In this work we have selected as an example the LHC-2 ring architecture of lamellar membranes of *R*. *molischianum* bacteria which are distributed randomly on top of a lamellar flat membrane [47]. Therefore, our artificial model of the LHC-2 architecture is a ring with an outer and inner diameter of 9 and 3.1 nm respectively, and a height of 5 nm (see Figure 4(a)), composed of a J-aggregate:PVA material as analysed above. This model will illustrate the nanophotonics of bio-mimetic ring structures containing densely packed J-aggregates, whether the ring structure consists of natural J-aggregates in a protein scaffold or synthetic cyanine J-aggregates in a PVA matrix. Note that our model aims to show whether polaritonic properties in the nanorings are possible. However, no potential anisotropies in the refractive index due to chromophores orientation are considered.

**Figure 5:**
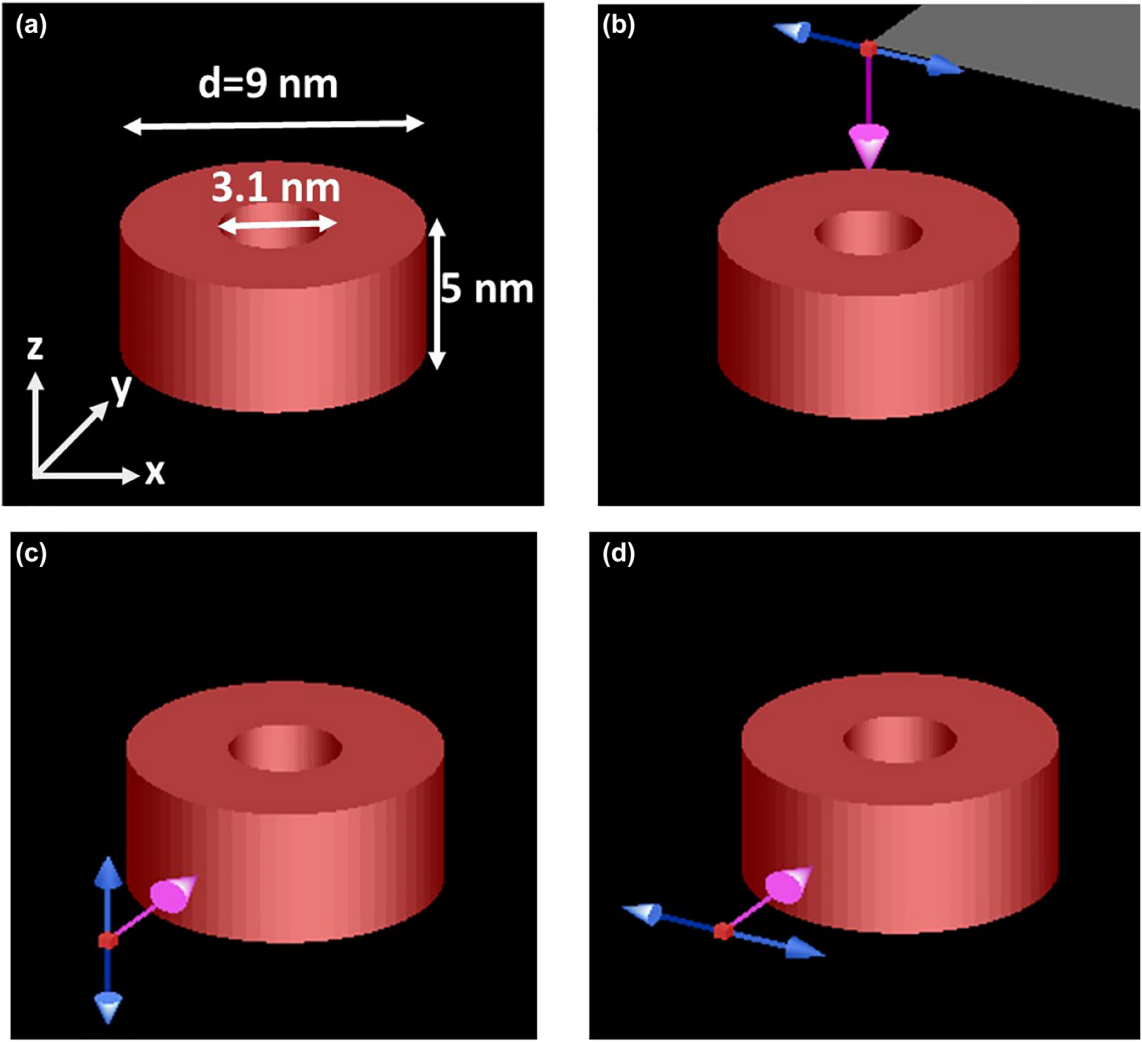
3D models of the bio-inspired nanostructures based on LH2 of *Rhodospirillum molischianum* bacteria used for simulations at different polarisation conditions. Incident light was set as linear polarised total-field scattered-field (TFSF) sources. The pink arrow indicates the propagation direction (wavevector) while the blue arrows indicate the direction of the polarisation vibration. Due to the geometry of the ring architectures three polarisations were set up to cover all potential combinations object-polarisation to determine the total absorption, scattering and extinction cross sections for un-polarised light (as under sun illumination).

The optical response and distribution of local electric fields have been estimated by finite difference time domain simulations using commercial software (Lumerical-Ansys). Rings of five different materials were simulated: the four J-aggregate:PVA materials studied above, and a fifth ring of PVA-only with no dyes, as a reference. The methodology and parameters used for the simulations are described in detail in Section 4 of the Supplementary (Figure 5). For the J-aggregate:PVA nanorings, the absorption cross section is four orders of magnitude larger than the scattering cross section due to the large imaginary part of the dielectric constant and the small size of the resonators (see Figure S6). Therefore, the absorption cross-section dominates the extinction cross-section. Figure 4 shows the extinction efficiency obtained for all J-aggregate: PVA nanorings and the reference. The reference shows a flat null response from 400 to 900 nm. However, the J-aggregate:PVA nanorings showed a clear extinction peak at shorter wavelengths than the absorption of the constituent J-aggregate but within the polaritonic band of each material (Table 1 and Figure 4), which is associated with a LSER. Importantly the extinction peak of the nanoring is centered at shorter wavelengths (higher energies) than the absorption peak of the monomeric dye (see Figure 4): this implies that rings may be capable of harvesting light at higher energies than the absorption peak of constituent dye through polaritonic trapping. This means that light-harvesting could be enhanced by polaritonic resonances associated with natural metamaterial nanostructures in the spectral range in which the constituent dyes themselves are less efficient at absorbing light directly. Furthermore, the proximity of the polaritonic resonance to the maximum of the dye absorption will increase the optical density of states at these wavelengths, enhancing the absorption and emission rate of the constituent dyes in the nanorings.

We now investigate the electric field distribution associated with LSER excitations of the J-aggregate:PVA LHC analogue nanorings, to provide insight into how the structure may control electromagnetic fields to improve light-harvesting. The distribution of the electric field is key to demonstrating firstly light confinement and secondly how the electric field is distributed, enhancing light–matter interactions in specific areas and coupling with other molecular entities. The LHCs of *R*. *molischianum* bacteria are distributed in the photosynthetic organelle within a lamellar photosynthetic membrane. Therefore, we considered two illumination incident conditions, parallel and perpendicular to the lamellar membrane. Figure 4(a) shows a three-dimensional representation of the modelled LHC nanoring. The yellow plane contains the lamellar membrane while the red plane is perpendicular to the lamellar membrane. We would like to remark that in natural photosynthetic membranes of *R*. *molischianum* bacteria, LHCs are closely packed within the membrane with a reaction centre located at the centre of some LHC nanorings.

**Figure 6:**
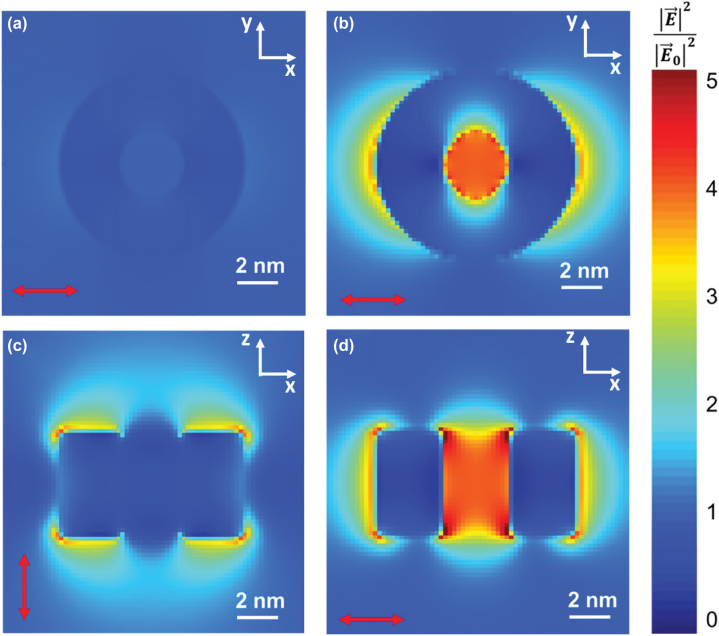
Two dimensional electric field intensity distributions at 609 nm for the 3D models of the bio-inspired nanostructures based on the LH2 of *Rhodospirillum molischianum* bacteria. Electric field intensity distribution in the *XY* plane crossing the nanoring in the middle for linear polarised light travelling in *Z* direction and polarised along *X* direction for (a) PVA-only nanoring and (b) J619 J-aggregate:PVA nanoring (oscillation direction indicated by the red arrows). Electric field intensity distribution at 609 nm in the *XZ* plane crossing in the middle a J619 J-aggregate:PVA nanoring for light linearly traveling in *Y* direction and polarised along the *Z* direction (c, see Figure 5(c)) and *X* direction (d, see Figure 5(d)).

Here we focus on LSERs of the J619 nanoring (similar results are obtained for J562 J587 and J798 J-aggregate:PVA materials, see Figures S8–S10). The Figure 6(b)–(d) shows the electric field intensity distribution at 609 nm (LSER peak for J619) for the reference nanoring (PVA only, Figure 6(a)) and a nanoring composed of J619 J-Aggregate:PVA material (Figure 6(b)–(d)). In the case of the reference nanoring, there is no local field enhancement, with a field intensity distribution close to one across the nanostructure (see Figure 4(a)). However, the electric field distribution is drastically different for the nanoring made of J619 J-aggregate:PVA. In this case, when the incident polarisation is in the plane of the lamellar membrane (Figure 6(b) and (d)), the electric field is concentrated at the centre and edges of the nanoring in the direction of the polarisation. Note that the reaction centre of a natural LHC is usually located at the centre of the nanoring structure. Therefore, our results suggest that the presence of J-aggregates in the natural nano-architecture may modify how light and excitons interact with the molecules of the reaction centre.

Furthermore, it is worth stressing that in a system formed by compact arrangements of several nanorings (similar to those observed in natural photosynthetic membranes) it is expected that the high electric filed concentration at the edges of each nanoring will promote coupling via evanescent waves between adjacent nanorings. This electromagnetic coupling between adjacent nanorings could promote exciton transport and energy transfer between them by polaritonic modes, as has been suggested in closed cavities [44, 49]. Further studies analysing complex distributions of LHCs nanorings including disorder are required for a more detailed analysis, though this is out of the scope of this article. Finally, it is remarkable how the optical response is strongly dependent on polarisation. While incident polarisation in the plane of the lamellar membrane concentrates the electric field in the centre of the nanoring and its lateral edges, when the polarisation is perpendicular to the lamellar membrane, the electric field at the centre is zero, and high field intensities are found at the top and bottom interfaces of the nanorings.

## 3 Conclusions

In this article, we present a new approach to metamaterials development inspired, firstly by how photosynthetic organelles are composed, and, secondly, by how their chromophores are arranged at the nanoscale. The relevance of these findings is twofold. Firstly, we have developed a complete library of densely packed supramolecular bulk materials based on synthetic photosynthetic chromophore antennas (J-aggregates) and a water-based polymer (PVA), with tunable optical properties from visible to infrared. Secondly, we studied how light interacts with ringed nanostructures with similar architecture to the natural ringed nanostructures found in the photosynthetic membrane of the purple bacteria *R*. *molischianum* for rings composed of our synthetic J-aggregate:PVA materials. Our results show that all of the studied J-aggregate:PVA materials have a strongly dispersive optical response creating highly reflective materials that support surface exciton polaritons. Therefore, all modelled nanostructures showed subwavelength confinement of light concentrating the optical field in the centre and edges of the nanorings through local surface exciton resonances. This demonstrates how densely packed dyes in synthetic nanostructures, similar to those found in natural systems, can create light confinement and field enhancement through electromagnetic polaritonic resonances, similar to plasmonics. Similarities between both the optical and excitonic properties of the natural and synthetic materials suggest that exciton polariton resonances could play a role in photosynthesis in purple bacteria. Furthermore, the specific features of these resonances show that they could be exploited to improve light-harvesting efficiency by broadening the wavelength response of constituent dyes while aiding energy transfer both to the reaction centre and between nanorings, through control and enhancement of the local electric field. Further studies on natural, larger scale distributions of LHCs in photosynthetic organisms composed of red- and blue-shifted aggregates are required to determine if through evolution organisms have developed nanophotonic strategies to optimise energy capture and exciton transport within photosynthetic organelles using excitonic metamaterials. Therefore, with this work, we would like to offer a new perspective for the development of novel organic metamaterial platforms and structures, taking inspiration from nature across length scales: from the molecular scale, passing through the mesoscale, towards well-defined large area metamaterial structures. The polymeric library and the suggested bio-inspired metamaterial building block can open the door to new metamaterial design strategies based on a polaritonic all-organic platform for light trapping and exciton transport. Potential experimental implementations of similar nanostructures could be based on the combination of molecular aggregates with colloidal and/or DNA-origami scaffolds [50–52].

## 4 Methods

### 4.1 Chemicals

All dyes were purchased from Few Chemicals:

– J562: (5-Phenyl-2-[2-[[5-phenyl-3-(4-sulfobutyl)-3H-benzoxazol-2-ylidene]-methyl]-but-1-enyl]-3-(4-sulfobutyl)-benzoxazolium hydroxide.
– J587: (5,6-Dichloro-2-[[5,6-dichloro-1-ethyl-3-(4-sulfobutyl)-benzimidazol-2-ylidene]-propenyl]-1-ethyl-3-(4-sulfobutyl)-benzimidazolium hydroxide.
– J619: (5-Chloro-2-[2-[5-chloro-3-(4-sulfobutyl)-3H-benzothiazol-2-ylidenemethyl]-but-1-enyl]-3-(4-sulfobutyl)-benzothiazol-3-ium hydroxide.
– J798 (: 5-Chloro-2-[5-[5-chloro-3-(4-sulfobutyl)-3H-benzothiazol-2-ylidene]-3-phenyl-penta-1,3-dienyl]-3-(4-sulfobutyl)-benzothiazol-3-ium hydroxide.
– PVA (Poly(vinyl alcohol), Mw 85,000–124,000, 99+% hydrolysed) was purchased in Sigma Aldrich.
– All water solutions were prepared with Milli Q-water.
– All ethanol solutions were prepared with ethanol synthesis grade.

*Preparation of PVA and cyanine water solutions*. The 6 wt% aqueous PVA solution was prepared under stirring at 90 °C for 1 h. The 25 mM solution of each dye was made under gentle stirring for several hours until a homogeneous solution of J-aggregates was reached.

*Deposition of J-aggregate*:*PVA films*. The J-aggregate:PVA films were obtained by spin-coating at 1000 rpm on top of a cover glass. Previously the deposition, the cover glass substrates were bathed overnight in 37 wt% HCl before rinsing in deionised water and drying with an air hose to obtain a hydrophilic surface.

*Unpolarised reflectance measurements*. Unpolarised reflectance spectra at six equally spaced angles of incidence between 45 and 70° were measured using a J.A. Woollam RC2 ellipsometer. Scotch tape was applied to the underside of each sample during measurement to provide an index-matched, rough bottom surface eliminating reflections from the bottom of the sample.

*P-polarised and s-polarised reflectance under Kretschmann prism-coupling configuration*. In this work, we used a tungsten-halogen white light lamp covering UV–vis–NIR spectral range for illumination. The optics consisted of a high numerical aperture Nikon plan APO 100× with NA = 1.45 mounted on a Nikon Ti2 microscope body. A fiber-coupled 2000+ Ocean Optics (Dunedin, USA) spectrometer was used. The prism coupling condition was obtained by illuminating from the glass side of each sample using an immersion oil objective. The calibration of the angular response is described in detail in Section 3 of the Supplementary information.

## Supplementary Material

Supplementary Material Details
