# Supplementary material for: Bio-inspired building blocks for all-organic metamaterials from visible to near-infrared
Source: Nanophotonics. 2023 Jan 20;12(2):307–18. doi: 10.1515/nanoph-2022-0690 (PMC11501215; doi:10.1515/nanoph-2022-0690)
Supplement: Supplementary file 1 — Supplementary Material Details [file j_nanoph-2022-0690_suppl.pdf]

Samuel Thomas Holder, Carla Estévez-Varela, Isabel Pastoriza-Santos, Martin Lopez-Garcia, Ruth Oulton and Sara Núñez-Sánchez

Bio-inspired building blocks for all-organic metamaterials from visible to near-infrared.

Supplementary Information

1 Chemical structure of the monomers of the polaritonic library

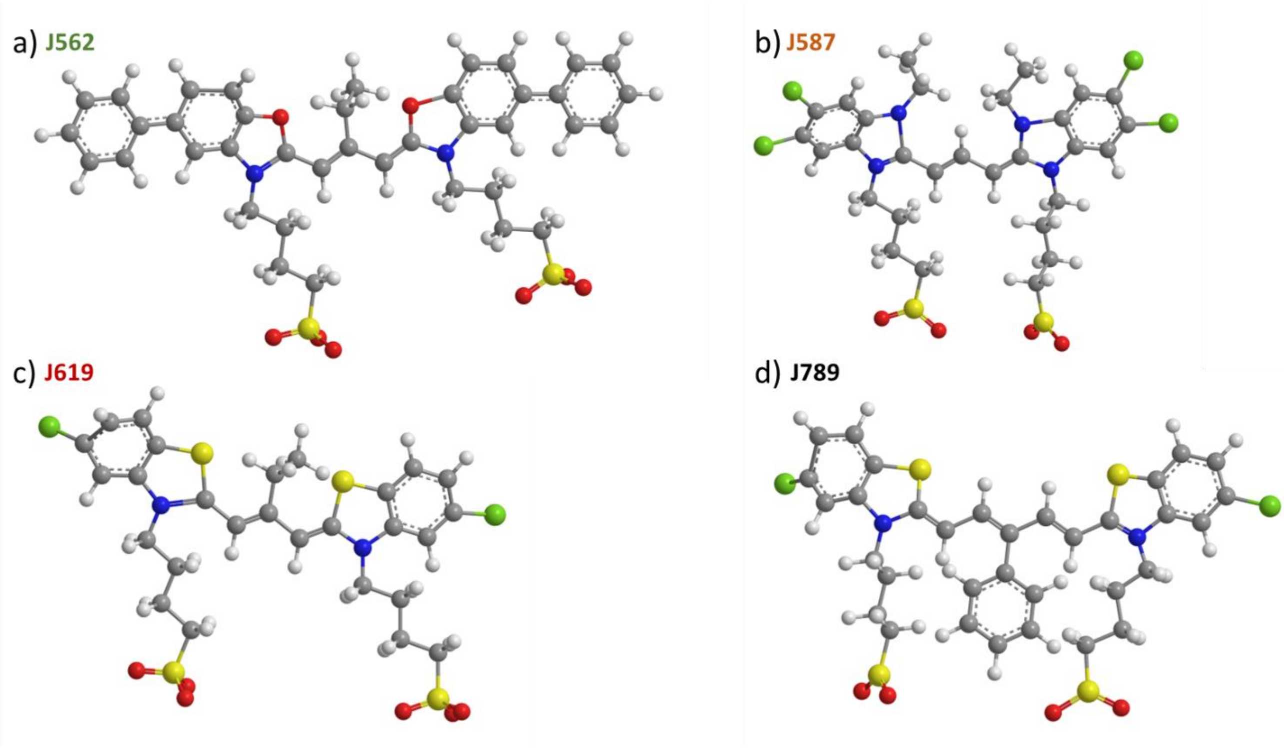

Figure S1: Chemical structure of monomers of (a) 5-Phenyl-2-[2-[[5-phenyl-3-(4-sulfobutyl)-3H-benzoxazol-2-ylidene]-methyl]-but-1-enyl]-3-(4-sulfobutyl)-benzoxazolium hydroxide (named J562) , (b) 5,6-Dichloro-2-[[5,6-dichloro-1-ethyl-3-(4-sulfobutyl)-benzimidazol-2-ylidene]-propenyl]-1-ethyl-3-(4-sulfobutyl)-benzimidazolium hydroxide (named J587), (c) 5-Chloro-2-[2-[5-chloro-3-(4-sulfobutyl)-3H-benzothiazol-2-ylidenemethyl]-but-1-enyl]-3-(4-sulfobutyl)-benzothiazol-3-ium hydroxide (named J619) and (d) 5-Chloro-2-[5-[5-chloro-3-(4-sulfobutyl)-3H-benzothiazol-2-ylidene]-3-phenyl-penta-1,3-dienyl]-3-(4-sulfobutyl)-benzothiazol-3-ium hydroxide (named J798).

2 AFM measurements and analysis

2.1 Determination of thin film thickness.

The thickness of the samples was determined by Atomic Force Microscopy (AFM, Agilent 5420 in tapping mode). Samples were scratched in a line using a flat clean room tweezer in several positions along with the sample. The thickness is estimated from the difference between the channel and the top of the film. Choosing two strips rather than averaging the whole image has two advantages: anomalous features such as dirt can be avoided, and the levelling of the image can be checked: both regions should give the same film thickness. Within these strips, which run left to right and are marked by white dotted lines in Figure S2, areas corresponding to the bare substrate and within 10μm the step edge are chosen by eye, and the average height of these areas is calculated. This gives an estimate of the substrate height. The same method is applied to the film area within the same strip to give an estimate of the film height. The difference in these average height values gives an estimate of film thickness, which is reported in table 1. The error on this thickness estimate is calculated by considering the independent height estimates that are averaged to estimate the substrate and film heights. The uncertainty in the film thickness estimate is given by the variance of these mean

values. The standard deviation of the mean of N independent samples, σ_N, for samples from a normal distribution with standard deviation σ, is

$$\sigma_N = \frac{\sigma}{\sqrt{N}}.$$

Not every measured point in these AFM images can be considered an independent height sample. The scanning of the atomic force microscope tip across the sample introduces correlations between subsequent height samples. Here these correlations have a characteristic length of around 1μm or five pixels. To get a lower bound on the number of independent height samples measured, N, we divided the total number of averaged points by 25, to impose a 1μm square pixel size. These N values together with the measured standard deviation of heights (σ), for substrate and film areas, gives the uncertainty in the height estimate assigned to each area via equation 3.2. These uncertainties are combined to give the uncertainty of the thickness estimate (table 1).

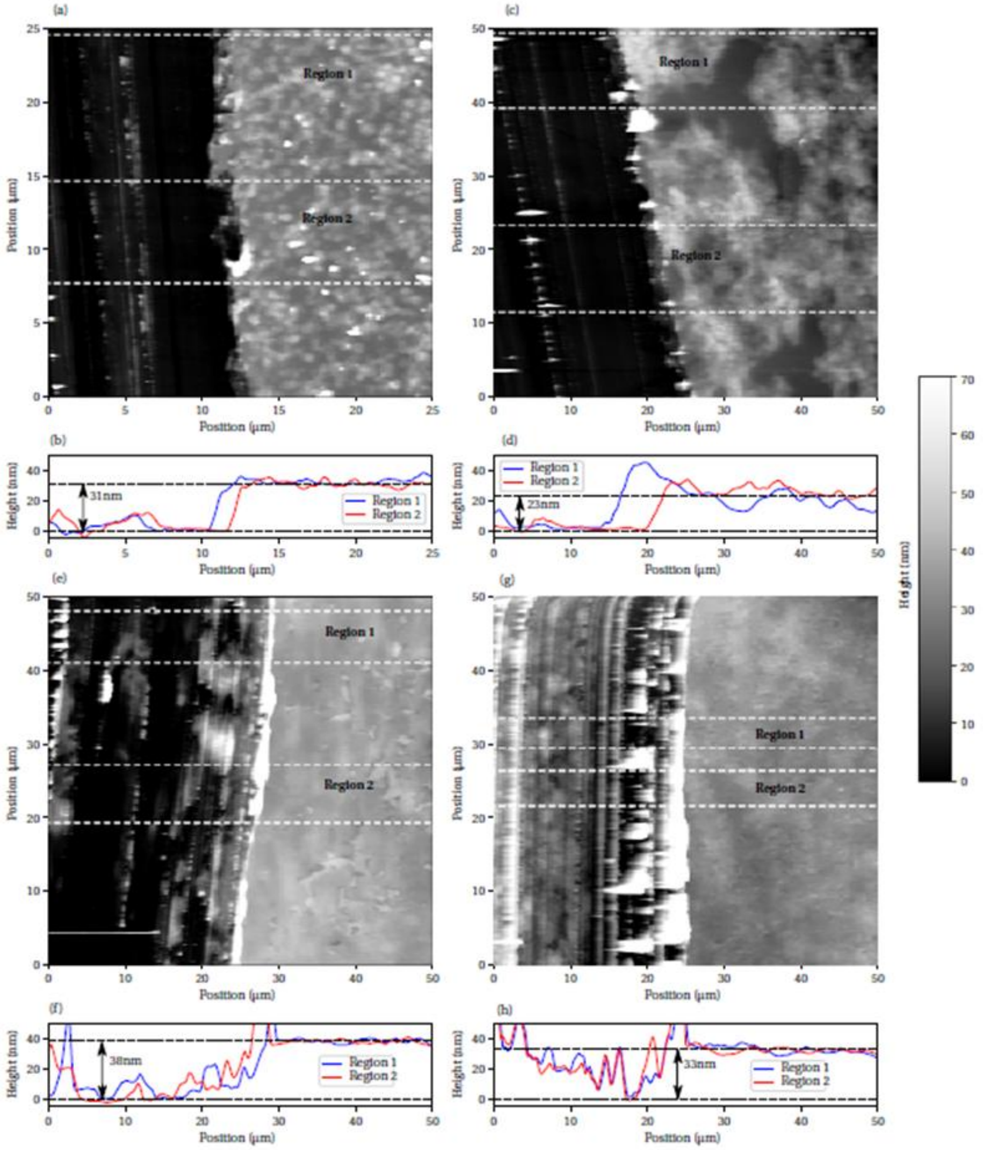

Figure S2: Atomic force microscope images of a substrate to film step in our J-aggregate and PVA films: (a) J562, (b) J587, (c) J619 and (d) J798. Each AFM image is marked with regions 1 and 2: the average profile of each of these strips is plotted below each AFM image. The substrate and film heights are marked with dotted red lines. Note that the J562 image is of a smaller (25 μm by 25μm) area.

2.2 Determination of surface roughness.

Surface roughness can modify the optical response of a material. Surface roughness can be quantified as the standard deviation (σ) of the distribution of measured surface heights (S):

$$\sigma^2 = \langle (\langle S \rangle - S)^2 \rangle$$

Note that σ is the conventional notation for the standard deviation of the measured surface heights, or equivalently the root-mean-square roughness. The standard deviation σ quantifies the width of the distribution of surface heights. Note that this simple metric does not use information about the height difference between adjacent points on the surface, and hence surfaces with very different textures can have the same roughness. However, σ is useful for estimating how big an effect the surface roughness will have on the optical response of a thin film. In table 1 we report the roughness of each of our J-aggregate and PVA thin films, based on the Figure S3 images where the pixel spacing (10nm) and the tip radius (<10nm) are comparable, meaning that the measured height image is close to the actual surface profile with only features below 10nm size being smoothed out. The statistical uncertainty of the measured σ values is given by

$$\sigma_\sigma^2 = \sigma \frac{1}{\sqrt{N}}$$

where N is the number of independent height samples used to calculate σ. Here, N is not identical to the number of pixels in the image. Instead, independent height samples are those separated by at least the correlation length of the surface. [1]

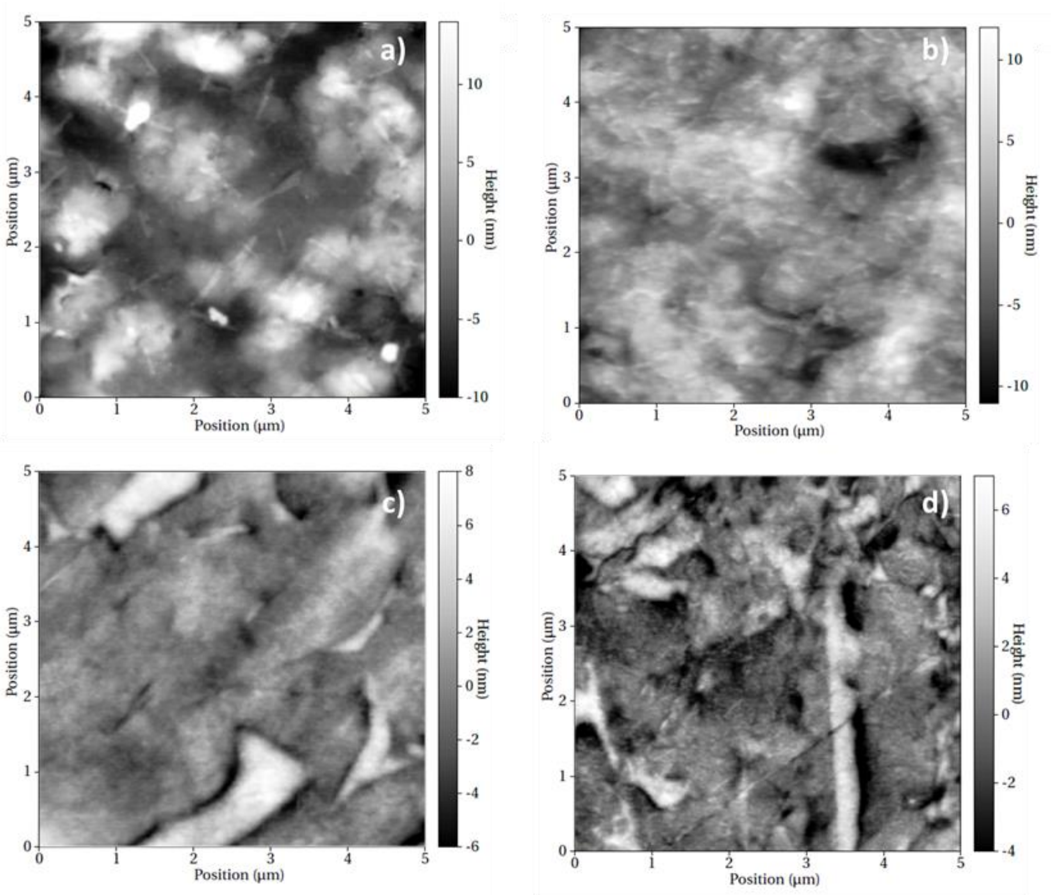

Figure S3. Height AFM images of the J-aggregate:PVA films of (a) J562, (b) J587, (c) J619, (d) J798 dye.

3 Fourier imaging spectroscopy under Kretschmann prism-coupling configuration.

A microscope cannot be used with a prism to make an angular reflectance measurement, as the short focal length objective lens requires for the sample to be brought close to the lens focussed on its surface. However, the Kretschmann prism-coupling configuration can be realised without a prism using an oil-immersion objective lens and a thin film sample deposited on a glass microscope coverslip (Figure S4). In this implementation, the high index region from which light is incident on the sample is formed by three different media: the bottom spherical lens of the oil-immersion objective, the immersion oil, and the glass substrate.

The samples were under critical illumination by a fibre-coupled tungsten lamp giving broadband illumination. The Fourier plane of the oil-immersion objective was imaged onto the tip of a scanning fibre which scans the Fourier plane along a straight line intersecting the centre of the Fourier plane, with each position corresponding to a different angle of incidence. The fibre output was coupled to a spectrometer allowing a broad wavelength range to be measured at each angle of incidence. The measured polarization was determined by the relative orientation of the measured line on the Fourier plane and a polarizer in the illumination path, to obtain either the p- or s-polarised reflectance.

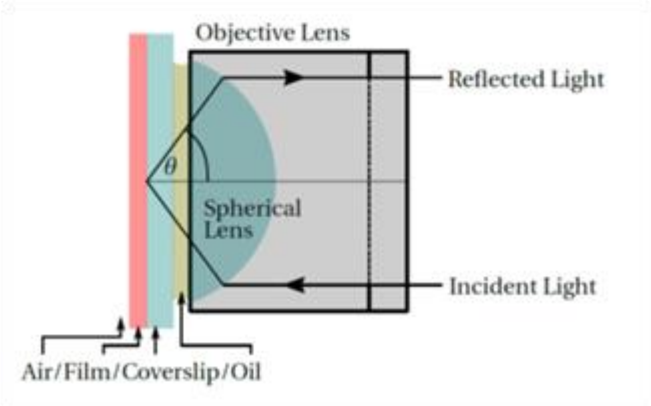

Figure S4: Scheme of the implementation of the Kretschmann prism-coupling configuration using an oil immersion objective lens.

To calibrate the angles on the image of the k-space obtained in the back focal plane of the objective, we used a coverslip as equivalent to the response of a prism with the refractive index of the glass (1.46). For angles of incidence smaller than the critical angle of a glass prism almost all the light pass through the interface glass/air and we should observe a minimum in reflectance. However, for angles larger that the critical angle, all the light is reflected due to matching total internal reflection conditions. Therefore, in the k-space, the response of a prism should be a black circle whose radius is defined by the total internal reflection condition (see Figure S5). The critical angle occurs when the x-component of the wavevector of light in the prism can no longer be matched to the x-component of the wavevector of light in the air above the prism. Moreover, to take a spectra reference of the source, we used a silver mirror as a reference with a flat reflection band in the visible with a reflectance close to a 100%. Figure S5 shows the reflectance obtained from a cover glass (equivalent of a prism) calibrated for the (a) scanning fibre.

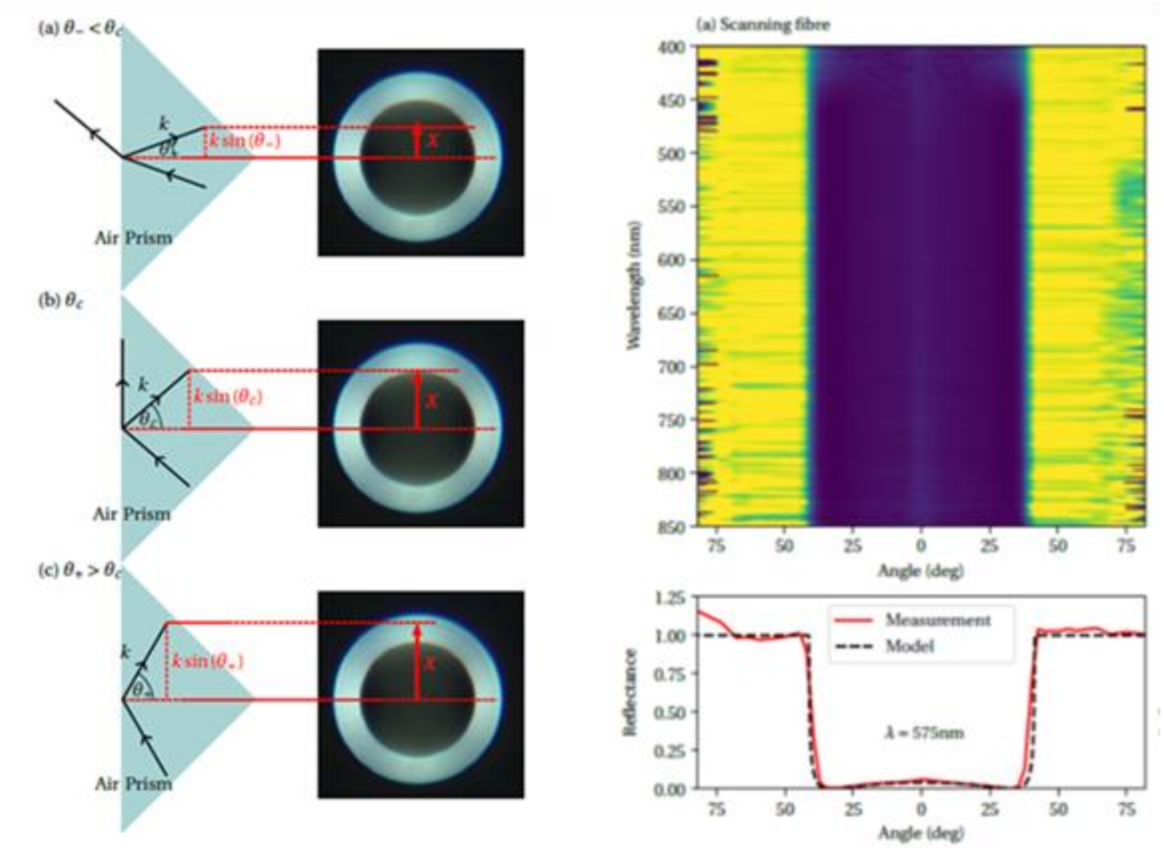

Figure S5: Reflectance from a prism at a range of angles, and the corresponding points on the observed camera image of the Fourier plane at three different angles: (a) below the critical angle, (b) at the critical angle and (c) above the critical angle. Reflectance of p-polarised light from a glass coverslip in the Kretschmann configuration, using different spectroscopic detection methods: (a) scanning fibre. The bottom panels show a cross-section through the above images at wavelength 575nm.

4 Defined objects and boundary conditions on the electromagnetics simulation set-up.

The optical response of nanoring structures with external diameter of 9 nm, an inner diameter of 3.1 nm and a height of 5 nm were obtained by FDTD simulations using commercial Lumerical software (Figure 4). These simulations have been performed for five different nanorings. Four of them will be composed by the optical properties of the four J-aggregate:PVA polymers. The fifth nanoring structure will be composed by just a dielectric polymeric matrix with a constant refractive index of 1.46 like PVA. This will be the reference for a LH2 ring architecture composed just by proteins without any chromophore. In all the cases we have considered the optical properties of the surrounding medium similar to the water with a refractive index of 1.33 as the natural light-harvesting nanostructures are embedded in an aqueous biological media. [2]

4.1 Estimation of absorption, scattering and extinction cross-sections for non-symmetric three-dimensional (3D) objects.

The absorption and scattering cross-sections were estimated by the combination total-field scattered-field (TFSF) source which injects a linear polarized plane wave with a finite span and a cross-section analysis group. The cross-section analysis group estimated the net power flowing through a 3D-field monitor with a box shape enclosing the nanoring normalized to the source intensity. In the case of the cross-section analysis group located inside the TFSF source we obtained the absorption cross-section and, in the case of the cross-section analysis group located outside the TFSF source we obtained the scattering cross-sections. All data is normalized to nanoring surface to obtain the cross-section efficiency per particle (equation S1).

$$\sigma_{particle} = \sigma_{total} / A_{ring}$$ equation S1

Because the cross sections are associated to three dimensional (3D) calculations, the relative orientation of incident polarized light with the object can play a role in how light is absorbed and scattered. In the case of spherical nanoparticles, the object is symmetric in the three dimensions, therefore it can be estimated by just by the cross sections for one polarization. However, the nanorings have a rotational symmetry in the vertical direction as cylinders. Therefore, the total cross sections were calculated considering all possible relative orientations of incident light and the nanorings. Figure 4 from manuscript shows the three different polarizations that we have considered for our calculations. The total cross sections were established by the average of all polarizations considering all the potential orientations and symmetries within the 3D-field monitor box (Figure 4, equation S2).

$$\sigma_{total} = \sigma_{polarization1} * 2\ (sides) + \sigma_{polarization2} * \frac{4\ (sides)}{2\ (polarizations)} + \sigma_{polarization3} * \frac{4\ (sides)}{2\ (polarizations)}$$,equation S2.

The Figure S6 shows the total absorption and the total scattering cross sections for the four J-aggregate:PVA nanorings and the reference nanoring without chromophores. The reference nanoring shows no-absorption and a flat extinction cross. However, the J-aggregate:PVA nanorings shown a clear absorption and scattering peaks at shorter wavelengths than the absorption of the bulk material which can be associated to a local surface exciton resonances.

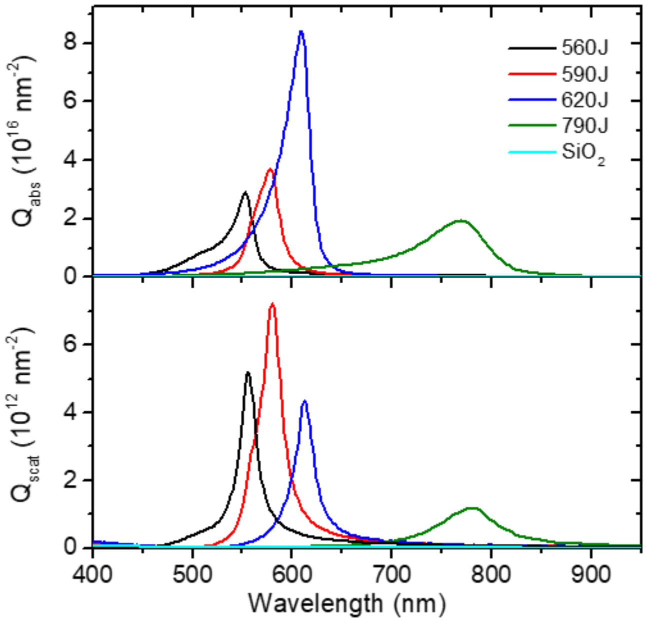

Figure S6. a) Absorption and b) scattering cross sections obtained for nanorings mimicking LH2 ring architecture of lamellar membranes of *Rhodospirillum molischianum* with the optical properties obtained for the bulk J-aggregate polymer films. Absorption cross-sections are peaked at 554, 578, 609 and 770 nm for J562, J587, J619 and J798 dyes, respectively. Scattering cross sections are peaked at 556, 580, 612 and 780 nm for J562, J587, J619 and J798 dyes, respectively.

The results shown in Figure 4.b shows the total extinction efficiency which is the extinction cross section normalized by the nanoring area (see equation S3).

$$\sigma_{extinction,\ total} = \sigma_{absorption,total} + \sigma_{scattering,total} \Rightarrow Q_{extinction} = \sigma_{extinction,total} / Area_{nanoring}$$ equation S3.

4.2 Local electric field distribution for perpendicular illumination to the lamellar membrane and polarization contained in the plane.

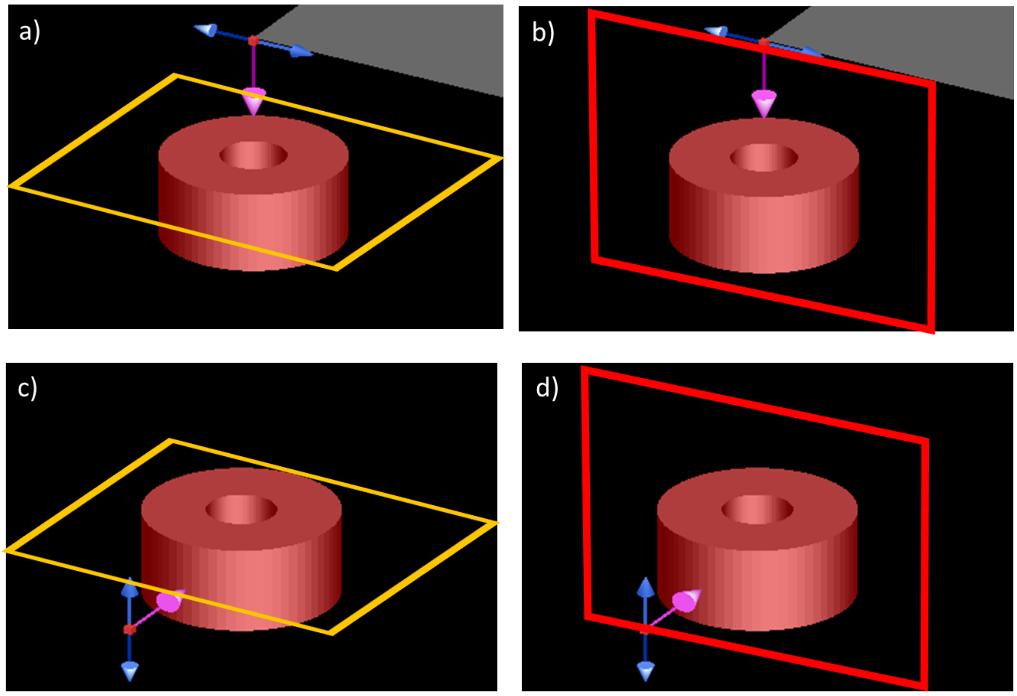

Figure S7.- a-b) Configuration of simulation with the light travelling perpendicular to the membrane. c-d) Configuration with the light travelling parallel to the membrane. The red and yellow squares are the planes where we have estimated the distribution of the local field intensities.

LH1 and LH2 ring complexes are ring architectural structures supported by a lamellar membrane located at the bottom of XY plane of the Figure S7. The yellow and red rectangle are the two planes where we have simulated the electric field intensity distribution for all the nanorings.

4.3 Local electric field distributions for J562, J587 and J798 dyes at LSER peaks.

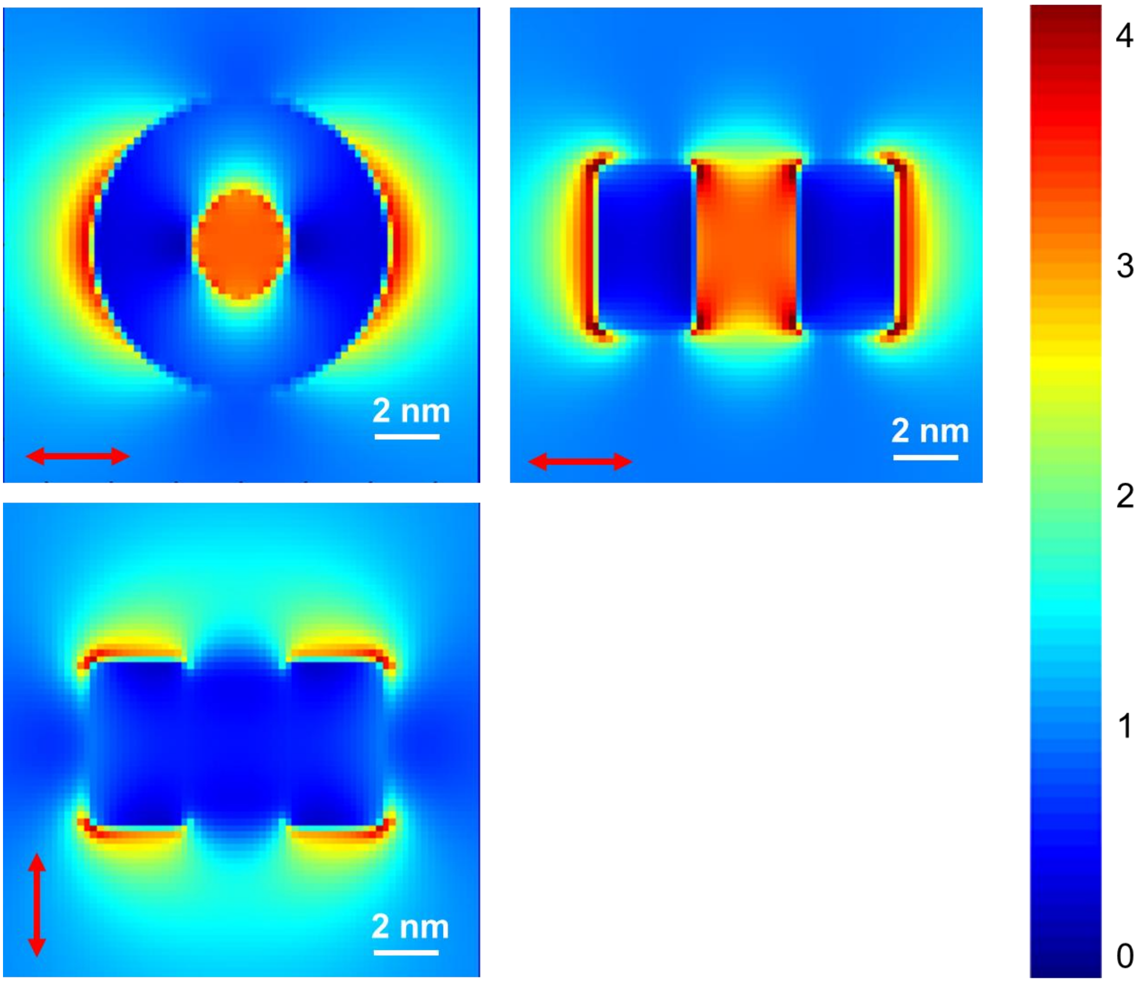

Figure S8.- Electric field intensity distribution for a nanoring composed by J-aggregate:PVA material of J562 dye at the LSER peak at 553 nm. Light linear polarized travelling towards the plane. Polarization indicated by red arrows.

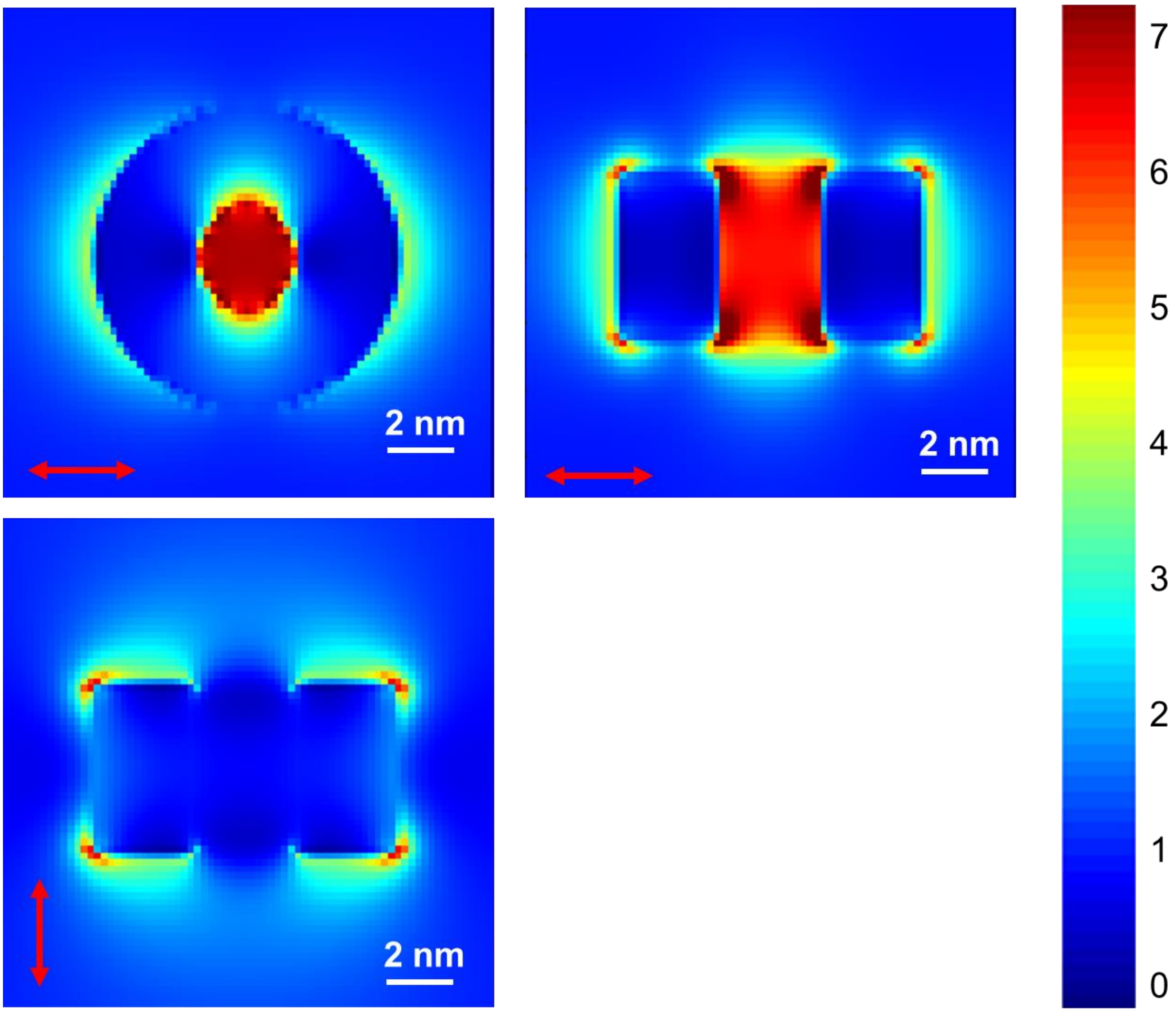

Figure S9.- Electric field intensity distribution for a nanoring composed by J-aggregate:PVA material of J587 dye at the LSER peak at 578 nm. Light linear polarized travelling towards the plane. Polarization indicated by red arrows.

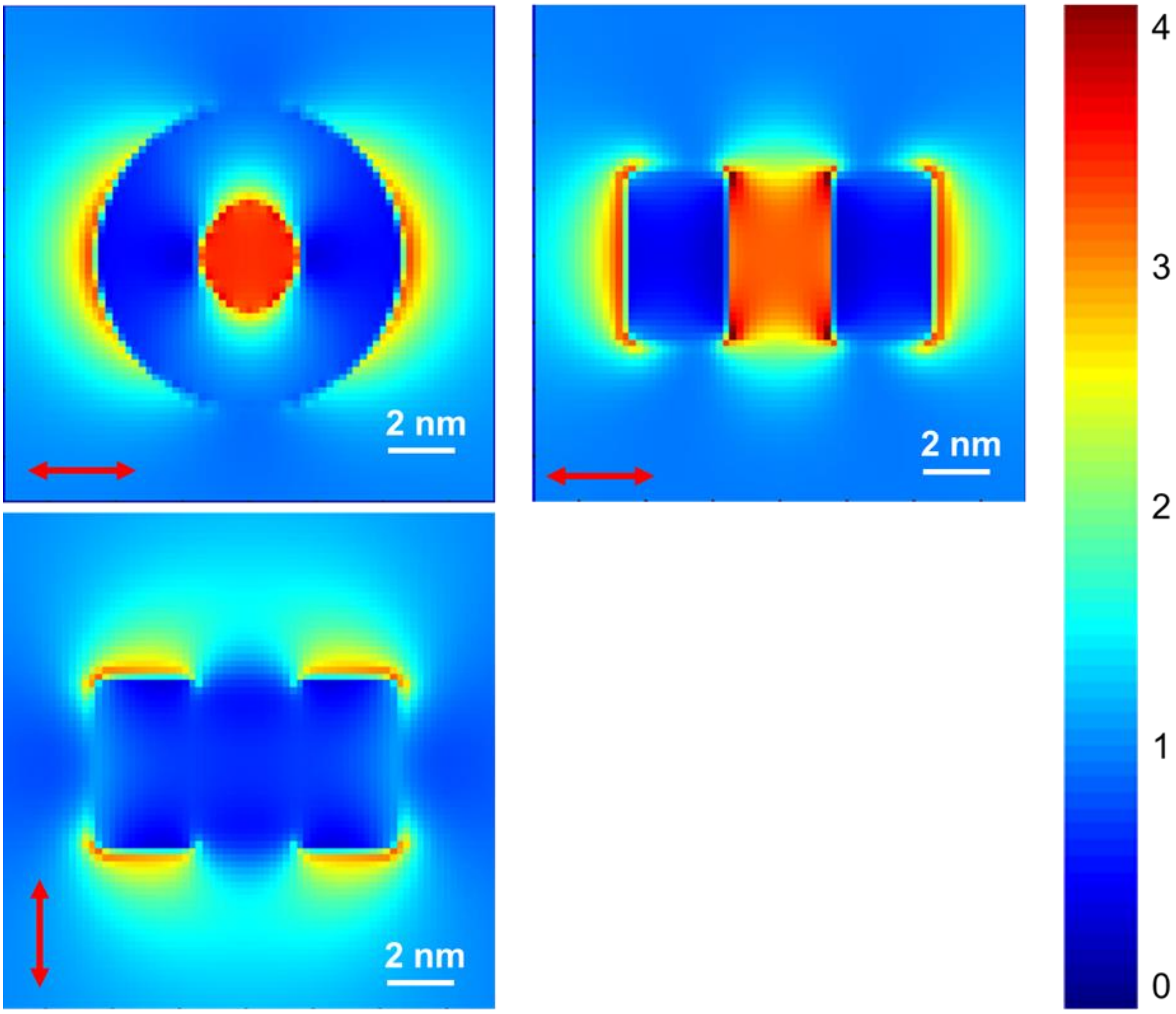

Figure S10.- Electric field intensity distribution for a nanoring composed by J-aggregate:PVA material of J798 dye at the LSER peak at 768 nm. Light linear polarized travelling towards the plane. Polarization indicated by red arrows.

5 REFERENCES

[1] C. A. Mack, *Uncertainty in Roughness Measurements: Putting Error Bars on Line-Edge Roughness*, J. Micro/Nanolithography, MEMS, MOEMS **16**, 10501 (2017).

[2] M. A. Castillo, W. P. Wardley, and M. Lopez-Garcia, *Light-Dependent Morphological Changes Can Tune Light Absorption in Iridescent Plant Chloroplasts: A Numerical Study Using Biologically Realistic Data*, ACS Photonics **8**, 1058 (2021).
